# Functional outcome improvement and surgical time reduction in a single‐surgeon consecutive case series of hip arthroscopy for femoroacetabular impingement: A minimum 5 years follow‐up study

**DOI:** 10.1002/jeo2.70022

**Published:** 2025-01-22

**Authors:** Enrico Tassinari, Filippo Caternicchia, Mattia D. Rosa, Francesco Castagnini, Edoardo Angeletti, Valentina Fantoni, Irene Tampieri, Andrea Burla, Stefano Zaffagnini

**Affiliations:** ^1^ Clinica Ortopedica E Traumatologica II IRCCS Istituto Ortopedico Rizzoli Bologna Italy; ^2^ Ortopedia I, IRCCS Policlinico San Donato, San Donato Milanese Milano Italy; ^3^ Ortopedia‐Traumatologia e Chirurgia protesica e dei reimpianti d'anca e di ginocchio IRCCS Istituto Ortopedico Rizzoli Bologna Italy

**Keywords:** conversion, experience, femoroacetabular impingement, labrum

## Abstract

**Purpose:**

The learning curve of a single surgeon performing hip arthroscopy is reported to be steep, but, to date, the inflection point after which procedures are more successful is still unknown. The aim of this study was to design a learning curve focused on clinical outcomes, complications and revision/conversion rates.

**Methods:**

Seventy‐one hip arthroscopies performed for femoroacetabular impingement (FAI) by a single surgeon, with a minimum follow‐up of 5 years, were considered. Demographics, intraarticular findings and operative and traction time were detailed. HOOS score, subjective outcomes, 30‐day complication rates, complication rates, revision arthroscopies and conversions to hip arthroplasty were recorded.

**Results:**

The mean follow‐up was 7.5 ± 1.8 years (range: 5–11). The progression of the learning curve implied a reduction in surgical time (*r*: −0.847), traction time (*r*: −0.806) and postoperative outcomes (*r* = 0.444). When the procedures were divided into three consecutive groups (25 vs. 25 vs. 21 procedures) or two consecutive groups (36 vs. 35 cases), the first group had a higher occurrence of 30‐day complications (*p* = 0.002 and *p* = 0.025, respectively) and the last group experienced a significant amelioration in terms of HOOS score between the preoperative and the postoperative condition (*p* < 0.001 and *p* = 0.018).

**Conclusions:**

The inflection point of the hip arthroscopy learning curve is between 25/36 procedures. The first arthroscopies were impacted by higher complications and lower clinical results but no higher rates of revision and conversion to arthroplasty.

**Level of Evidence:**

Level IV.

**Abbreviations**
BMI: body mass index
FAI: femoral‐acetabular impingement
HOOS: Hip disability and Osteoarthritis Outcome Score
PROMs: patient‐reported outcomes

## INTRODUCTION

Hip arthroscopy is an effective and innovative procedure, with rapidly expanding indications [9, 12]. However, although there is a steady increase in hip arthroscopy procedures, the literature agrees about the steep learning curve of this procedure [11]. To date, there is no agreement about the number of procedures required to significantly reduce complications and conversions to hip arthroplasty: the reported values ranged from 20 to 500 [6, 14]. This great variability is mainly due to different research methodologies and the dissimilar definitions of the learning curve: some studies focused on clinical outcomes (clinical scores, complications), and few investigated the revision and conversion rates [4, 14]. Moreover, the enrolled populations were heterogeneous, involving large registry‐based cohorts and short‐term single‐centre case series [9, 14, 21].

Therefore, a retrospective study was designed in order to evaluate a series of consecutive hip arthroscopies carried out by a single operator to treat intraarticular pathology, with a minimum follow‐up of 5 years. The aim of this study was to determine the learning curve for hip arthroscopy and in particular the inflection point defined as the point of the learning curve where we can observe stable clinical results (assessing the long‐term outcomes of a medical procedure in which a patient's medical condition has either improved or remained the same over a period of time, without any significant worsening or relapse), minimal complication rates and stable revision/conversion rates.

## MATERIALS AND METHODS

The local institutional review board approved the study (580/2020/Oss/IOR). A consecutive series of hip arthroscopies performed for femoroacetabular impingement (FAI) by a single surgeon (Enrico Tassinari) were retrospectively selected from the hospital database. The procedures were chronologically ordered, from the very first performed procedure to the last one. Before the first hip arthroscopy, the single surgeon had previous experience with knee arthroscopy and joint arthroplasty procedures. Diagnosis of FAI was determined according to the clinical findings (pain, limited articular excursion, provocative manoeuvres) and radiographic assessment in antero‐posterior and axial views (herniation pits, pistol grip deformity, crossover sign, centre‐edge angle >39° and/or alpha angle >50°) [10]. For every case, a 3‐ to 6‐month course of conservative treatments, including physiotherapy, was undertaken before arthroscopic procedure.

The inclusion criteria were hip arthroscopy for FAI performed by a single surgeon, a minimum follow‐up of 5 years and patients' adherence to the study protocol. The exclusion criteria were hip arthroscopy for intra‐articular pathologies not FAI related (loose bodies, synovium pathologies and internal snapping syndrome), extra‐articular pathologies, surgeons other than the designed one and inadequate follow‐up/medical records. For every patient, data about demographics (age, sex, side, American Society of Anesthesiologists classification and body mass index [BMI]) were collected. FAI was graded as cam, pincer or mixed [18]. The osteoarthritis degree according to Tonnis was recorded using the preoperative pelvis X‐rays [3]. Using the surgical report, data about the intra‐articular pathologies (labral tear, chondral damage, type of impingement) and their treatment were collected, as well as the surgical time (from skin incision to skin closure) and the traction time. The patients were clinically assessed at the last follow‐up using the Hip Disability and Osteoarthritis Outcome Score (HOOS) score [17]. The patients were also asked to rate their satisfaction about the procedure (four degrees: completely satisfied, quite satisfied, moderately satisfied and unsatisfied). The perioperative complications (30 days) were collected, as well as the complications that occurred during the other check‐ups after surgery. Arthroscopic revisions and conversions to hip resurfacing/arthroplasty were recorded.

### Statistical analysis

The quantitative data were expressed as mean, standard deviation and range of minimum and maximum values, whereas the qualitative variables were defined as absolute frequencies and percentages. The Pearson test was adopted to express the linear relationship between variables. The Spearman test was used to assess the correlations between continuous variables. The Student test was adopted to compare the mean values between two cohorts. The variability between groups was assessed using the analysis of variance (ANOVA) test. The Fisher exact test was used to test the frequency distribution for dichotomous variables in small samples. All the statistical analysis was performed using the IBM SPSS software (version 25). The threshold for significance is *p* = 0.05.

## RESULTS

About 71 patients met the inclusion criteria: 42 males (59.2%) and 29 females (40.8%). Thirty‐seven hips were left‐sided (54.9%) and 34 were right‐sided (45.1%). The mean BMI was 22.6 ± 2.2 kg/m^2^ (range: 18.7–26.5). The mean follow‐up achieved was 7.5 ± 1.8 years (range: 5–11). The type of FAI, the osteoarthritis degree and the intra‐articular findings are detailed in Table 1. Seventy‐one labral lesions were observed (100% of patients): in 77.5% of cases, the lesion was treated by debridement. Fifteen labrums were sutured (21.1%). Nine cases of labrum instability were stabilized by electrocoagulation (12.7%). Microfractures were performed in 53 patients (74.6%). Acetabular and femoral osteoplasty was performed in 33 cases (46.5%), while femoral osteoplasty alone was performed in 33 cases (46.5%). In four cases (5.6%), no bone osteoplasty procedure was performed. In one case (1.5%), acetabular osteoplasty alone was performed.

**Table 1:** Demographics and intraoperative findings were recorded.

Study demographics		
Femoroacetabular impingement type		
CAM	29	
Pincer	5	
Mixed	37	
Osteoarthritis degree according to Tonnis		
Tonnis grade 0	20	
Tonnis grade 1	30	
Tonnis grade 2	21	
Tonnis grade 3	0	
Chondropathy according to Outerbridge		
I°	15 (21.12%)	
II°	26 (36.71%)	
III°	25 (35.2%)	
IV°	5 (7%)	
Labral lesions/Labrum instability	71 (100%)	

Eight postoperative complications (11.3%) were observed, of which seven were hypoesthesia of the pudendal nerve and one was hypoesthesia of the dorsal surface of the foot; the complications were correlated with traction time (Pearson test, *r* = 0.74). At the last follow‐up, osteoarthritis progression was observed in eight patients (11.3%), periarticular ossifications were recorded in one patient (1.5%), residual hip pain was present in one patient (1.5%) and joint effusion occurred in one case (1.5%). The progression of osteoarthritis was not correlated to preoperative chondral damage or osteoarthritis (Pearson test, *r* < 0.3).

**Figure 1:**
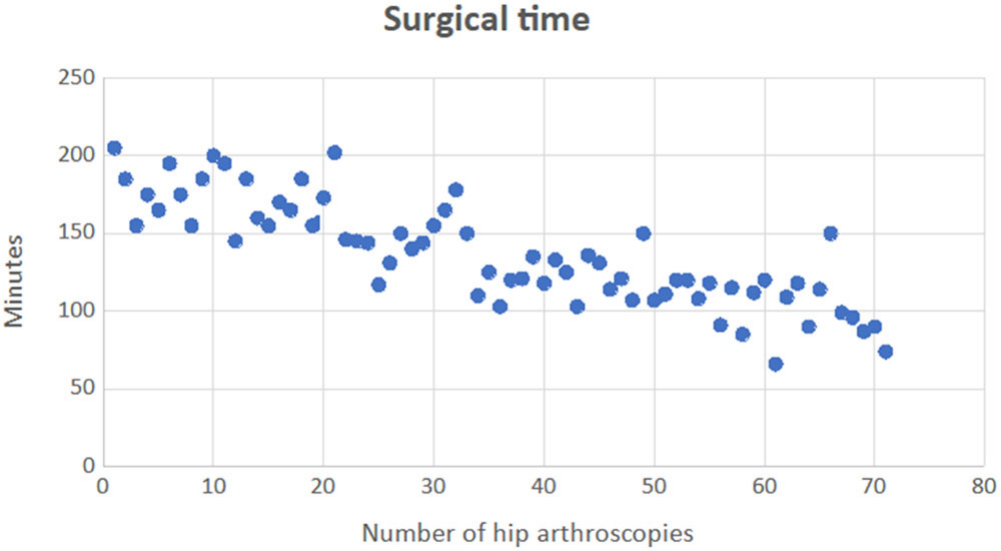
Significant reduction of surgical time as the learning curve progresses (Spearman test, *r* = −0.847).

**Figure 2:**
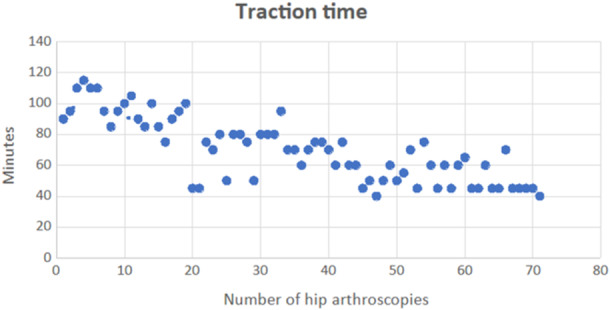
Significant reduction of traction time as the learning curve progresses (Spearman test, *r* = −0.806).

**Figure 3:**
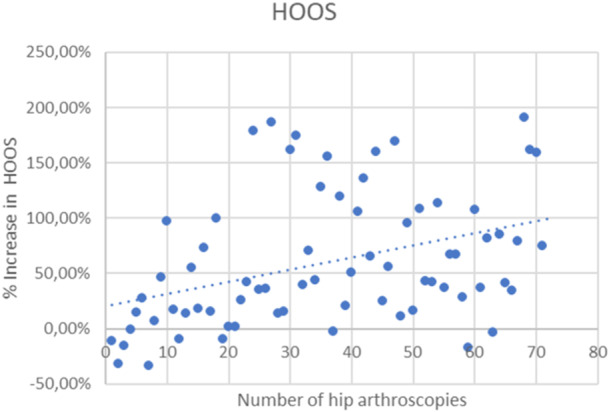
A progressive improvement in the Hip Disability and Osteoarthritis Outcome Score (HOOS) difference (*p* < 0.001) between pre‐ and postoperative conditions (in percentage) was observed as the number of arthroscopies performed by a single surgeon increased.

The mean final HOOS was 75.11 ± 18.6 points (range: 23, 75–100). There was a significant postoperative increase in HOOS clinical score (*p* < 0.001). At the last follow‐up, the arthroscopic procedure left nine patients (12.7%) poorly satisfied, 16 patients (22.5%) moderately satisfied, 21 patients (29.6%) quite satisfied and 25 (35.2%) patients completely satisfied. At a minimum follow‐up of 5 years, we recorded eight conversions to hip arthroplasty (11.3%) and five arthroscopic revisions (7%). As the learning curve progresses, a significant reduction of surgical time (Spearman test, *r* = −0.847) and traction time (Spearman test, *r* = −0.806) was observed (Figures 1 and 2); the postoperative HOOS score also significantly improved, with a lower correlation (Spearman test, *r* = 0.444). As the learning curve progressed over time, an increment in terms of HOOS score between preoperative and postoperative status was noticed (Figure 3).

The procedures were divided into three consecutive groups according to chronological order, from the first performed procedure to the last one: the first cohort included 25 patients, the second one included 25 patients and the third one included 21 patients. There were no differences among the three groups in terms of HOOS outcome (one‐way ANOVA test, *p* > 0.05), subjective outcomes (Fisher exact test, *p* > 0.05), postoperative complications (Fisher exact test, *p* > 0.05), revision rates (Fisher exact test, *p* > 0.05) and conversions to arthroplasty (Fisher exact test, *p* > 0.05). The first group had a higher occurrence of 30‐day complications (Fisher exact test, *p* = 0.002) and the last group experienced a significant amelioration in terms of HOOS score between the preoperative and the postoperative condition (one‐way ANOVA test, *p* < 0.001). When the 71 arthroscopies were chronologically segmented into two groups (36 vs. 35 cases), the first group had a higher incidence of 30‐day complications (Fisher exact test, *p* = 0.025) and the second group experienced a significant improvement in terms of HOOS score between the pre‐ and the postoperative condition (one‐way ANOVA test, *p* = 0.018). No differences were evident about HOOS outcome (one‐way ANOVA test, *p* > 0.05), subjective outcomes (Fisher exact test, *p* > 0.05), postoperative complications (Fisher exact test, *p* > 0.05), revision rates (Fisher exact test, *p* > 0.05) and conversions to arthroplasty (Fisher exact test, *p* > 0.05).

## DISCUSSION

The analysis of the first 71 consecutive hip arthroscopies performed by a single surgeon for FAI at a minimum follow‐up of 5 years showed that, as the number of arthroscopies increases, the surgical time and the traction time are reduced, with an improvement of clinical outcomes and the rate of 30‐day complications. No significant differences could be observed about patient‐reported outcomes. The inflection point was between 25 and 36 procedures: nevertheless, no impact on the conversion to total hip arthroplasty or revision rates was observed at a minimum follow‐up of 5 years.

The surgical technique of hip arthroscopy is complex and demanding, especially because of the hip joint depth and the large surrounding muscle groups limiting the manoeuvrability and the visibility [7, 13, 18, 23]. Thus, hip arthroscopy was associated with a steep learning curve [2]. A recent review of the literature identified a cutoff ranging from 20 to 500 cases before good and stable results could be achieved, in terms of surgical time, exposure to fluoroscopy, patient satisfaction and a reduced number of complications and re‐operations [7, 16]. However, many criticisms could be raised about these outcomes. Many studies provided an indirect measure of the learning curve, as exposure to fluoroscopy or surgical time; while both these indicators may give many hints about surgical abilities and experience, they remain two proxies, since these variable indicators do not necessarily reflect actual learning or the underlying cognitive processes involved in learning [10]. For example, traction time may be even longer when surgeons increase experience and may take on more complex cases or additional intra‐articular procedures [7]. As a matter of fact, Go et al. noticed that out of 15 studies included in a systematic review about the learning curve in hip arthroscopy, only four studies truly investigated a proficiency curve (using different outcomes and different methodologies) [7]. Konan et al. noticed a learning curve of 30 cases in a single‐surgeon nonhomogeneous case series of 100 cases at short‐term, defined as better subjective and objective clinical scores, time reduction and low complication rates [12]. In a single surgeon case series of 40 hip arthroscopies, Lee et al. highlighted that after 30 procedures, failures achieved an acceptable rate and there was a time reduction [14]. Mehta et al. performed a registry study evaluating conversion and reoperation rates at 5 years: a learning curve of more than 500 procedures was found [16]. Considering the very different study methodologies and the different proficiency outcomes, providing data about patient‐reported outcomes (PROMs), scores or re‐operation/conversion rates seems a correct method. However, there is still a great variability among cutoffs of PROMs and clinical score values: many authors report that the first 20–30 arthroscopies are deemed to be inferior results [4, 5, 7, 19, 20, 22]. Our report observed that 25/36 cases may be a good approximation; after this threshold, better clinical outcomes and shorter operative times could be observed. Moreover, similarly to four studies out of ten providing data about complication rates, a lower rate of complications was observed after 25/35 arthroscopies [6, 7, 12, 20]. Once again, the involved studies used different assessments and provided different thresholds, impeding a reliable comparison. Invariably, most of the complications in the literature and in the present paper were neurological in origin.

Only five studies out of 15 about learning curve in hip arthroscopy focused on reoperation/conversion rates; specifically, only four papers provided data about the rate of conversions to hip replacement [7]. In these studies, re‐intervention and conversion rates ranged from 12.2%−22.5% in the first cases to 1.5%−3.7% in the last cases [7, 15, 19]. We noticed similar outcomes, even if a longer follow‐up was provided in the present report. As a matter of fact, none of the four above‐mentioned studies described a minimum mid‐term follow‐up (of at least 5 years) [1, 7]. Surprisingly, we observed no differences in terms of reoperation/conversion rates when the first cases were compared to the latter cases. This is in line with some case series comparing chronologically ordered arthroscopies at short‐term [7].

The paper has many limits; among them are the single surgeon selection, the modest number of patients, the nonhomogeneous selection of patients (consecutive), different intraarticular pathologies, different degrees of cartilage wear and the lack of exposure to fluoroscopy. On the other hand, the main strong point of this paper is the single‐surgeon consecutive series of patients at a minimum mid‐term follow‐up of 5 years [1]; the present study is the first in the available literature with a mid‐term follow‐up done by a single surgeon with mild experience of knee arthroscopy but no experience of hip arthroscopy at that time. This point may provide a true perspective on the complications and the conversion/revision rates, which are among the most important outcomes to validate the learning curve. Plus, the learning curve was represented as a proficiency curve with a steady increment of the HOOS score delta; also, dichotomous comparisons were provided in the case of categorical variables [7, 8].

## CONCLUSION

Considering the available literature and the present work, 25/36 arthroscopies may be a possible inflection point after whom clinical outcomes and, mostly, complication rates may lower, even at mid‐term follow‐up. However, it seems that no difference is evident in terms of revision and conversion rates at a minimum 5‐year follow‐up. Considering that the first arthroscopies are more prone to complications and inferior outcomes, adequate supervision should be counselled for the first 30 cases.

## AUTHOR CONTRIBUTIONS

All authors contributed equally.

## CONFLICT OF INTEREST STATEMENT

The authors declare no conflict of interest.

## ETHICS STATEMENT

All procedures performed in the current study were in accordance with the 1964 Helsinki Declaration and its later amendments. The study design was approved by the institutional review board of Istituto Ortopedico Rizzoli, study protocol 580/2020/Oss/IOR. Written informed consent was obtained from all individual participants included in the study.

## Data Availability

The authors confirm that the data supporting the findings of this study are available within the article and its supplementary materials.
